# Expanding HIV Testing Efforts in Concentrated Epidemic Settings: A Population-Based Survey from Rural Vietnam

**DOI:** 10.1371/journal.pone.0016017

**Published:** 2011-01-11

**Authors:** Anastasia Pharris, Nguyen Thi Kim Chuc, Carol Tishelman, Ruairí Brugha, Nguyen Phuong Hoa, Anna Thorson

**Affiliations:** 1 Division of Global Health/IHCAR, Department of Public Health Sciences, Karolinska Institutet, Stockholm, Sweden; 2 Health Systems Research Project, Hanoi Medical University, Hanoi, Vietnam; 3 Department of Learning, Informatics, Medical Management and Ethics (LIME), Medical Management Center, Karolinska Institutet, Stockholm, Sweden; 4 Population Health Sciences Division, Department of Epidemiology and Public Health, Royal College of Surgeons Ireland, Dublin, Ireland; University of Cape Town, South Africa

## Abstract

**Background:**

To improve HIV prevention and care programs, it is important to understand the uptake of HIV testing and to identify population segments in need of increased HIV testing. This is particularly crucial in countries with concentrated HIV epidemics, where HIV prevalence continues to rise in the general population. This study analyzes determinants of HIV testing in a rural Vietnamese population in order to identify potential access barriers and areas for promoting HIV testing services.

**Methods:**

A population-based cross-sectional survey of 1874 randomly sampled adults was linked to pregnancy, migration and economic cohort data from a demographic surveillance site (DSS). Multivariate logistic regression analysis was used to determine which factors were associated with having tested for HIV.

**Results:**

The age-adjusted prevalence of ever-testing for HIV was 7.6%; however 79% of those who reported feeling at-risk of contracting HIV had never tested. In multivariate analysis, younger age (aOR 1.85, 95% CI 1.14–3.01), higher economic status (aOR 3.4, 95% CI 2.21–5.22), and semi-urban residence (aOR 2.37, 95% CI 1.53–3.66) were associated with having been tested for HIV. HIV testing rates did not differ between women of reproductive age who had recently been pregnant and those who had not.

**Conclusions:**

We found low testing uptake (6%) among pregnant women despite an existing prevention of mother-to-child HIV testing policy, and lower-than-expected testing among persons who felt that they were at-risk of HIV. Poverty and residence in a more geographically remote location were associated with less HIV testing. In addition to current HIV testing strategies focusing on high-risk groups, we recommend targeting HIV testing in concentrated HIV epidemic settings to focus on a scaled-up provision of antenatal testing. Additional recommendations include removing financial and geographic access barriers to client-initiated testing, and encouraging provider-initiated testing of those who believe that they are at-risk of HIV.

## Introduction

Many countries with concentrated HIV epidemics are currently grappling with developing long-term strategies for HIV prevention and surveillance within their communities, particularly outside urban areas and epidemic “hot spots” [1]. In Vietnam, 73% of the population of 86 million live in rural areas [2], [3]. HIV control efforts in concentrated epidemic settings, including Vietnam, have focused on high-risk groups in urban areas such as intravenous drug users (IDU), commercial sex workers (CSW), and more recently, men who have sex with men (MSM) [1], [4].

The risk group-focused approach can be difficult to translate into practice, as individuals tend not to think of themselves as members of risk groups to the extent that medical and public health professionals do, particularly related to risk that is associated with highly stigmatized and illegal behaviors such as sex work or drug use. Additionally, persons at-risk for HIV may not fall into traditionally defined high-risk groups. In fact, Vietnam's Ministry of Health estimated that in 2007, 31% of all new HIV infections were among “low-risk” men while an additional 13% and 14% were among “low-risk” rural and urban women, respectively [5]. Many of the infections among low-risk men and women are due to HIV transmission to clients of sex workers and, in turn, to clients' female partners, as well as to the partners of former or current IDU [4]. The national adult HIV prevalence in Vietnam is estimated to be 0.44%, which is slightly higher than the regional South and South-East Asia estimate of 0.3%, and there are indications that HIV prevalence is increasing among the general population [4], [5], [6], [7].

Together, testing and counseling for HIV form an important HIV prevention measure and a gateway to treatment for persons living with HIV [8],[9]. HIV testing is generally either initiated by individuals who would like to be tested (client-initiated HIV testing) or by health care providers (provider-initiated testing). Provider-initiated testing can be “opt-out” where routine HIV testing is performed unless the patient refuses, or “opt-in” where patients are asked and tested if they choose to do so [10].

In Vietnam's National Strategy for HIV/AIDS Prevention and Control (2005–2010) [11], the goal for population-based HIV testing was to reach 50% of districts in all provinces by 2010. This strategy also entails the integration of HIV testing into maternal and family health services, including provider-initiated testing of all pregnant women who seek antenatal care (ANC) or delivery in public facilities (more than 90% of Vietnamese women attend ANC at least once during pregnancy) [11], [12]. The plan focuses especially on targeting services to most at-risk populations such as IDU and CSW, as well as to migrants and persons in remote areas. It also envisages combining HIV testing with the diagnosis and treatment of tuberculosis and sexually transmitted infections [11]. Throughout Vietnam, HIV testing is typically organized through district hospitals or, less commonly, private clinics rather than through specialized HIV testing centers.

While information on testing rates and gaps are not routinely available, it is thought that the majority of those in need of HIV testing and treatment have not yet been reached by HIV programs [6], [13]. Evidence from Vietnam does indicate that persons with HIV often present very late for testing, in an advanced stage of illness [14]. This translates into lower survival on antiretroviral therapy and a higher risk of transmission of HIV to sexual and injecting partners prior to learning one's HIV status [15], [16]. Reasons for presenting late for testing have not been clearly described in Vietnam, however in other settings they include HIV-related stigma and barriers to accessing HIV testing including testing cost, location, perceived quality, and perceived confidentiality [17], [18], [19].

While the HIV case-finding capacity of the health system in Vietnam is limited, additional HIV testing strategies are necessary in order to identify a high proportion of HIV infections early and to mitigate the impact of these infections through treatment and enhanced prevention. A better understanding is needed of the factors associated with those who access HIV testing in a timely way, those who delay and those who do not seek testing. In this population-based survey we aimed to analyze determinants of HIV testing in a rural Vietnamese population in order to make recommendations for additional targeted HIV testing in settings with concentrated HIV epidemics, such as Vietnam.

## Methods

### Study setting

This study was conducted in Bavi district, a largely rural farming district located 60 kilometers northwest of Hanoi. HIV testing services have been available at Bavi district hospital since 1998, and antiretroviral treatment for HIV, which became available in Hanoi in 2005, was extended to a town located about 25 kilometers from Bavi district in 2007. A national plan to conduct opt-out HIV testing of all women attending antenatal care was outlined in 2005. In addition, provider-initiated HIV testing without the patient's knowledge does occur, e.g. prior to major surgery. Otherwise, the HIV testing that occurs is typically client- or provider-initiated for suspected AIDS cases. In 2006, patients paid about 30,000 Vietnam Dong (US $1.80) out-of-pocket for HIV testing. While no accurate HIV prevalence estimates exist, the reported HIV rate in Bavi district in 2007 was 0.12%, calculated based on all reported HIV positive cases. (Personal communication, Bavi District Preventative Health Director, 25^th^ Sept 2008, A Pharris).

Size estimates of populations most at-risk for HIV such as IDU, CSW, and MSM in this district have not been studied and are uncertain. The tuberculosis rate in this area was estimated at 100/100,000, although under-detection is common with a case-detection rate of 39% in men and 12% in women [20]. Employment migration between the study area and the higher HIV-prevalence areas of Hanoi City and Quang Ninh province is common and constitutes a possible bridge between this rural community and higher prevalence areas.

### Study population

This study was conducted in April-May 2007 within the rural demographic surveillance site (DSS) FilaBavi located within Bavi district. For the DSS sample, Bavi district (population approximately 262,000) was divided into 352 clusters and stratified into four geographical areas: lowland, highland, island, and mountainous areas. Highland areas are generally nearer to the main roads and health services. Seventy-one of these clusters were then sampled, with probability of inclusion proportional to the size of the cluster's population, and were included in the DSS. Since its inception in 1999, FilaBavi DSS has conducted quarterly surveys of vital events in its sample of 12,818 households, including 50,456 individuals (2007 population numbers). In the present population-based survey, two-stage random cluster sampling was used to identify 1874 adults (18-60 years) stratified by age and sex from the adult DSS sample, in a random sample of 46 of the 71 clusters.

### Ethics statement

Ethical permission was granted by Vietnam's Hanoi Medical University. In line with the ethical approval given and following the informed consent procedures used within the DSS since 1999, the study's purpose was carefully explained to each individual and verbal informed consent was received prior to each individual's inclusion in the study.

### Data collection

Face-to-face interviews with participants were conducted using a structured questionnaire administered in a private area, in or close to respondents' homes. Interviews took place during routine quarterly DSS data collection and were carried out by 46 female DSS surveyors who received study-specific training. Routine DSS data collection consists of interviewing the head of household about new vital events in the household using structured questions. For this study, one adult within the household was randomly sampled, given information about the study and interviewed in person if s/he consented to participate.

The questionnaire, which included structured questions about HIV-related knowledge, attitudes, and testing behavior, was pre-tested and revised prior to training surveyors and commencing data collection. Six trained supervisors and a field coordinator supervised data collection and provided quality assurance.

### Data analysis

EpiData version 3.1 (EpiData Association, Odense, Denmark) was used for data entry and STATA version 9.0 (Stata Corporation, College Station, TX, USA) was used for data processing and analysis. The data collected in this survey were linked to household socioeconomic, pregnancy, and migration data for the DSS cohort. Geographic areas were dichotomized into the more urbanized highland area and the more remote non-highland areas (including mountainous, lowland, and island areas). Economic status was determined by principal components analysis (PCA) for household assets and dichotomized into the poorest 60% and the least poor 40% [21]. Women aged 18 to 45 years were classified into recently pregnant (those who had been pregnant, regardless of pregnancy outcome, during the 3 years prior to data collection or who were pregnant at the time of data collection) and not recently pregnant (women aged 18 to 45 who had not been pregnant during the three years prior to data collection). Long-term migration was defined as having left the district for 3 months or more during the 7 years prior to the survey; those who had not left or migrated for shorter than 3 months were classified as non-migrants.

Pearson's Chi-square and odds ratios with 95% confidence intervals were employed to test for associations between HIV testing and demographic and HIV risk variables. HIV testing rates for men and women were age-adjusted for the population structure of 18–60 year olds for the entire DSS sample based on 2007 population numbers. A multivariate logistic regression model was constructed to identify factors independently associated with the dependent variable: HIV testing. Independent variables found to be significant in bivariate analysis at a level of p<0.25 were included in the model and removed using a backwards stepwise method. Independent variables were tested and only one interaction term involving two variables –“feels at risk for HIV” and “plans to test for HIV in the coming year” - was statistically significant and included in the final model. The goodness of fit for the model was tested using the Hosmer-Lemeshow method and through examining receiver operating characteristic (ROC) curve plots [22]. The final area under the ROC curve was 0.829, meeting criteria for good model discrimination [23].

## Results

### Sample background characteristics

Ninety-eight percent of the 1874 respondents in this sample (basic socio-demographic characteristics of the study sample are shown in Table 1) reported having heard of HIV, with television the most frequent source of HIV information, followed by radio, magazines or newspapers, and friends or relatives. As seen in Table 2, only 38% of respondents, and significantly fewer women than men (34% versus 42%, p<0.001), reported having enough information to protect themselves from contracting HIV. Over one-third of all respondents reported not knowing whom they could ask about HIV (Table 2). Ten percent of women and a higher (14%) percentage of men reported feeling at personal risk for HIV (p = 0.002, Table 2). Forty-one percent of respondents stated that they knew someone with HIV and 90% reported that they believed HIV to be a problem in their community (Table 2).

**Table 1:** Socio-demographic characteristics (n = 1874).

		Women	Men	Total
		n = 943	n = 931	n = 1874
		n (%)	n (%)	n (%)
**Age**	*Mean ± SD*	*37.4±11.7*	*37.4±11.9*	*37.4±11.8*
	18–29 years	313 (33)	304 (32)	617 (33)
	30–44 years	321 (34)	314 (34)	635 (34)
	45–60 years	309 (33)	313 (34)	622 (33)
**Education level**	Primary (≤6 years)	179 (19)	161 (17)	340 (18)
	Secondary (7–12 years)	676 (72)	664 (71)	1340 (72)
	Tertiary	88 (9)	106 (11)	194 (10)
**Economic status**	Poorest 60%	574 (61)	557 (60)	1131 (60)
	Least poor 40%	369 (39)	374 (40)	743 (40)
**Place of residence**	Lowland	178 (19)	167 (18)	345 (18)
	Highland	492 (52)	491 (53)	983 (52)
	Mountainous	250 (27)	252 (27)	502 (27)
	Island	22 (2)	21 (2)	43 (2)
**Long-term migration**	Left home for >3 months during last 7 years	84 (9)	149 (16)	233 (12)

Note: Totals for each variable are in some cases less than 1874. Economic status missing for 20 individuals; information on place of residence and long-term migration missing for 1 of the total sample.

**Table 2:** HIV testing and HIV risk perceptions (n = 1874).

	Women	Men	Total
	n = 943	n = 931	n = 1874
	n (%)	n (%)	n (%)
Doesn't know how to prevent HIV transmission*	89 (9)	50 (5)	139 (7)
Doesn't know who to ask about HIV	361 (38)	319 (34)	680 (36)
Believes s/he has enough information to protect self from HIV*	318 (34)	391 (42)	709 (38)
Believes HIV is a big problem in (his/her) community	836 (89)	846 (91)	1682 (90)
Knows someone with HIV	389 (41)	376 (40)	765 (41)
Feels at risk for HIV *	90 (10)	131 (14)	221 (12)
HIV tested in past#	58 (6)	77 (8)	135 (7)
Age-adjusted HIV testing rate (%)	(6.4%)	(8.9%)	(7.6%)
Plans HIV test (this year)	30 (3)	26 (3)	56 (3)

*Significant difference between women and men at p<.05.

#The denominator for HIV tested in past is 1818 (total sample minus 39 non-responses and 17 persons who were unsure whether they might have tested for HIV).

### HIV Testing

The age-adjusted prevalence of having been tested for HIV was 7.6%, with a slightly higher proportion of men (8.9%) having been tested than women (6.4%, p = 0.073). A higher proportion of younger persons reported having had an HIV test: 9.2% of 18–29 year olds compared to 4.6% of those ages 45–60 (data not shown). The 217 women who had been pregnant or recently given birth had a slightly lower HIV testing rate (6%) than the female non-recently pregnant population under 45 years of age (7.5%, p = 0.48).

Over three-fourths of those reporting feeling at risk for HIV and 89% of those reporting knowing someone with HIV had never been tested for HIV (Table 3). Very few people reported plans for future HIV testing (3%). In a logistic regression model, factors found to be associated with HIV testing were: younger age (aOR 1.85, 95% CI 1.14–3.01), higher economic status (aOR 3.4, 95% CI 2.21–5.22), highland residence (aOR 2.37, 95% CI 1.53–3.66), long-term migration out of the district (aOR 2.7, 95% CI 1.62–4.47), feeling that one has enough information about HIV (aOR 1.97, 95% CI 1.3–2.98), feeling at-risk for HIV (aOR 3.81, 95% CI 2.42–6.0), listing condoms as an HIV prevention method (aOR 1.65, 95% CI 1.06–2.58), knowing someone with HIV (aOR 1.78, 95% CI 1.17–2.7), and planning to test for HIV this year (aOR 12.0, 95% CI 5.10–28.28) (Table 3).

**Table 3:** Multivariate analysis of background characteristics and HIV testing (n = 1818*).

		Total not HIV tested (n = 1683)	Total HIV tested (n = 135)	Crude OR	Adjusted OR
		n (%)	n (%)	(95% CI)	(95% CI)**
**Age**	18–44 years	1107 (91)	107 (9)	1.99 (1.29–3.05)	1.85 (1.14–3.01)
	45–60 years	576 (95)	28 (5)	1.0	1.0
**Sex**	Male	825 (91.5)	77 (8.5)	1.38 (0.97–1.97)	-
	Female	858 (93.7)	58 (6.3)	1.0	-
**Education level**	Primary (≤6 yrs)	304 (94)	20 (6)	1.07 (0.64–1.77)	-
	Secondary (7–12 yrs)	1231 (94)	76 (6)	1.0	-
	Tertiary	148 (79)	39 (21)	4.27 (2.79–6.51)	-
**Economic status**	Poorest 60%	1048 (96)	44 (4)	1.0	1.0
	Least poor 40%	635 (87)	91 (13)	3.41 (2.35–4.96)	3.4 (2.21–5.22)
**Place of residence**	Highland area	858 (90)	94 (10)	2.2 (1.51–3.22)	2.37 (1.53–3.66)
	Non-highland area	825 (95)	41 (5)	1.0	1.0
**Long-term migration**	No	1485 (93)	104 (7)	1.0	1.0
	Yes	198 (86)	31 (14)	2.24 (1.46–3.43)	2.7 (1.62–4.47)
**Lists condoms as an HIV prevention method**	No	1360 (94)	88 (6)	1.0	1.0
	Yes	323 (87)	47 (13)	2.25 (1.55–3.3)	1.65 (1.06–2.58)
**Believes s/he has enough information about HIV**	No	1055 (95)	56 (5)	1.0	1.0
	Yes	628 (89)	79 (11)	2.37 (1.66–3.38)	1.97 (1.3–2.98)
**Knows someone with HIV**	No/not sure	1003 (95)	53 (5)	1.0	1.0
	Yes	680 (89)	82 (11)	2.28 (1.59–3.27)	1.78 (1.17–2.7)
**Feels at-risk for HIV**	No	1500 (94)	88 (6)	1.0	1.0
	Yes	174 (79)	47 (21)	4.6 (3.13–6.78)	3.81 (2.42–6.0)
**Plans HIV test this year**	No	1647 (93)	115 (7)	1.0	1.0
	Yes	36 (64)	20 (36)	7.96 (4.46–14.19)	12.0 (5.10–28.28)
**Recent pregnancy (Women <45 years, n = 617)**	No	370 (92.5)	30 (7.5)	1.0	-
	Yes	204 (94)	13 (6)	0.79 (0.40–1.54)	-

OR = odds ratio;

*Data on HIV testing was available for 1818 participants, plus additional missing values for “feels at-risk for HIV” (9).

**The final multivariate logistic model includes all variables with adjusted odds ratios listed plus an interaction term for feels at-risk x plans to test this year.

## Discussion

In this population-based survey, we found low HIV testing rates in the adult population, particularly among women who were or had recently been pregnant. Considerable differences in HIV testing across segments of this rural population were observed, with lower HIV testing reported among persons of lower economic status and more remote non-highland residence. We also found that 79% percent of those persons who believed that they were at-risk for HIV had never been HIV tested and that very few people, in general, had plans to test for HIV in the coming year. These results are discussed in terms of their implications for HIV testing within antenatal care settings, as well as for provider- and client-initiated HIV testing of the general population in concentrated HIV epidemic settings.

The low rate of testing in the antenatal care (ANC) setting in this Vietnamese rural population provides one indication that the uptake of prevention of mother-to-child (PMTCT) programs in this area was very low (6%) at the time of this study. In the Vietnam Population AIDS Indicator Survey (VPAIS) 2005, it was estimated that 10% of women nationally and 60% of women in Hanoi were tested for HIV during ANC [24], while 86% of a nationally representative sample of rural Thai women tested for HIV at ANC [25]. Although the scope of the present study was not to determine why ANC testing uptake was low, studies of PMTCT care from other settings in Vietnam have found a failure of health workers to offer HIV testing, possibly due to unclear guidelines for how to offer and pay for opt-out testing during ANC; fears of or actual breaches of confidentiality in HIV test result disclosure procedures; and, more generally, women's fear of the social stigma of living with an HIV diagnosis [10], [26], [27], [28]. Our results showed that women were more likely than men to report lacking information about HIV prevention and were less likely to know whom they could ask about HIV; they perhaps did not identify the ANC setting as a place where HIV knowledge or information could be obtained.

In another province in Northern Vietnam, Dinh et al [29] found that about half of women offered HIV testing during pregnancy did not accept it and an additional half of those who tested did not return for their results. Not testing for HIV was associated with believing that one was not at-risk, indicating that risk perceptions are low among pregnant women in Vietnam [5], [29]. However, HIV prevalence continues to increase among pregnant women, largely due to transmission from male sexual partners [4]. Women are not always aware of or willing to acknowledge and disclose their male partners' risk behaviors, leading to low risk perception and low uptake of HIV testing. When pregnant women are not tested for HIV, there is a risk of a “double miss” failing to identify those in need of antiretroviral therapy to reduce the risk of HIV transmission to the child in utero, as well as missing an opportunity to identify a woman eligible for antiretroviral therapy, thereby improving her chances of survival and reducing the risk of her child becoming orphaned [30].

In addition to lower-than-expected HIV testing among pregnant women, our results also point to rural residence as an access barrier to client-initiated HIV testing in this district. In this rural sample, 8.9% of men and 6.4% of women reported having ever had an HIV test as compared to the much higher rates of 25% reported among adults in urban Hanoi, Vietnam [24], [31] and 48% found in a national survey of sexual behavior in Thailand [32]. The lower rates of testing of persons in Bavi district compared to those in Hanoi (just 60 kilometers from parts of Bavi district), exemplify rural:urban inequities, which may be due to the lower availability of testing in rural areas, but may also be due to low uptake among those living in rural districts compared to those in Vietnam's major cities. Within our sample, there was lower HIV testing by persons living in more geographically remote non-highland areas within the district, perhaps because they lived further from the district hospital where HIV testing was offered. We also saw that persons who had migrated out of the district, usually to larger cities or the higher HIV-prevalence coastal provinces in the northeast of Vietnam, had higher rates of testing than those who had not migrated. Geographical barriers are well-documented in inhibiting access to health services, including HIV testing [33], [34]. With 75% of its population in rural areas, this points to a problem that Vietnam and many other countries have in serving those outside of major urban areas and suggests that strategies targeting these less urban areas must be strengthened to reduce access barriers to client-initiated HIV testing [6], [35], [36].

Our results indicate that lower socio-economic status may act as another barrier to client-initiated HIV testing, suggesting that HIV testing is accessed through out-of-pocket payments by those who can afford it. Even if cost-free testing is offered (in this district testing was not cost-free in 2007), private fee-for-service care is often preferred for diseases, like HIV, where high stigma fuels a desire for anonymity; this may limit access for the lowest income strata [37]. These findings are consistent with the 2005 VPAIS and have also been documented outside of Vietnam [24], [38]. In Vietnam, there is no evidence that HIV infection is associated with higher socio-economic status, as has been found in some settings with generalized HIV epidemics [39]. Organizing free HIV testing so that it is confidential and of high-quality will help to reduce the potential cost barrier to client-initiated HIV testing for poorer individuals. Towards this goal, there are reports that free HIV testing was beginning to be offered during antenatal care in the study district in late 2010.

In addition to HIV testing which is available in the antenatal care setting and freely and easily accessible to those who wish to access it, it is important that health care providers also screen patients for risk behaviors and initiate HIV testing when they believe that it is appropriate. Provider-initiated testing in Vietnam currently focuses on those patients who present very ill to outpatient or inpatient settings, and is part of the diagnosis of AIDS-related illnesses such as tuberculosis. Due to the high stigma associated with HIV and the close association between HIV, drug use and sex work, some providers might be hesitant to ask patients about HIV risk behaviors or fear interacting with patients with HIV [28], [40]. However, enhanced provider screening could lead to more HIV testing in the outpatient setting and more case-finding of early HIV infections. In the present study, the majority (79%) of patients who felt at-risk for HIV had never been tested for HIV. While their hesitance to do so may have been due to barriers to accessing care or because they did not wish to know their HIV status, confidential, high-quality provider-initiated counseling and testing might have enhanced the numbers of those who tested for HIV. HIV-related stigma among community members and providers likely hinders HIV testing uptake, but enhanced focus on training providers and encouraging more provider-initiated HIV testing could aid in the effort to de-stigmatize HIV and boost testing uptake [41].

In a concentrated HIV epidemic, universal HIV testing is neither an appropriate nor an optimal use of limited public health resources. However, it is crucial that those persons who are most likely to have HIV be encouraged to test and, if positive, given antiretroviral therapy and adequate support to prevent the further spread of HIV. In settings like Vietnam, targeting patients on the basis of risk group membership has not resulted in high proportions of persons being tested early in the course of their HIV infection. Therefore, where increasing HIV prevalence rates are seen among persons who are not classified in high-risk groups, additional strategies for case-finding of persons with HIV are necessary. The present study points to some key areas for enhanced planning and program implementation in concentrated HIV epidemic settings. These include antenatal care testing, removing barriers to client-initiated HIV testing, and enhanced focus on provider-initiated testing. While the ideal threshold for population-wide HIV testing must be based on the epidemiological context, there are areas for enhanced testing focus in low prevalence, concentrated HIV epidemic settings like Vietnam. In addition to a continued strong focus on HIV testing within IDU, CSW, CSW client, and MSM risk groups, an enhanced focus could be placed on high-quality and confidential: i) ANC testing of close to 100% of all pregnant women; ii) removal of access barriers to client-initiated testing by organizing the free provision of HIV testing accessible to persons in all geographic areas; and iii) enhanced provider-initiated testing of persons who believe that they are at-risk for HIV.

This study was conducted in 2007 and, since then, there have been changes in the highly dynamic field of HIV prevention and testing in this district in Vietnam. There are reports of increased training of health workers on HIV testing and enhanced attention to HIV testing in the antenatal setting. Still, stigma continues to be high and significant challenges to HIV testing remain. Our results illustrate factors associated with low uptake of HIV testing; however, low availability of HIV testing is likely to have contributed to low HIV testing rates in this study, particularly antenatal and client-initiated HIV testing. We did not study provider-related factors that could have affected respondents' ability to receive HIV testing and further study of this is necessary to determine whether a description and analysis of provider factors would need to precede an expansion in testing services. Studies of provider-related issues might be better undertaken through audits of HIV testing facilities, in which confidentiality and accessibility are also measured and described. We conducted our study in a demographic surveillance site, which was constructed in 1999 to be representative of a typical Vietnamese rural district in terms of socio-demographics and health status [42]. While changes since 1999 have led to parts of the district becoming semi-urban, we believe that the study area is still fairly representative of most of rural Vietnam and study results could be generalized, depending on local HIV epidemic contexts.

While our study has the strength of using population-based data, a limitation is its reliance on self-reported data. HIV testing experiences might be underreported due to recall bias and we did not obtain information on whether HIV test results were received. However, testing for a highly stigmatized disease like HIV is likely to be recalled. Also, due to the stigma attached to HIV, some individuals might have opted for non-reporting. To minimize this, we conducted the study in a private area, used interviewers who had trusted relationships with the respondents, and chose not to ask about individual risk behaviors so as to enhance patient openness to participate in the study. To further guide public health actions, future research should assess the community acceptability of asking about individual risk behaviors, perhaps though computer-assisted interview devices, as well as the possibility of offering HIV testing within the DSS framework in Vietnam.

The data presented here indicate areas for enhanced targeting of HIV testing in concentrated HIV epidemic settings, particularly focusing on the provision of antenatal HIV testing, the removal of financial and geographic access barriers to client-initiated testing, and on encouraging provider-initiated HIV testing among those who believe that they are at risk for HIV. This may be achieved in a rural concentrated HIV epidemic setting by offering free, high-quality, and confidential HIV testing which is easily accessible, while ensuring that provider-initiated, opt-out testing be offered to all pregnant women and those who believe that they might be at-risk for HIV. DSS sites provide a valuable mechanism for monitoring population HIV risk factors, HIV testing uptake, and possibly prevalence, both in the general population and in demographic and geographic sub-groups. Given the limited resources and competing health priorities in many low and middle-income countries with concentrated HIV epidemics, it is important that the potential for DSS-based research be fully utilized by policy makers and health researchers for HIV monitoring and planning.

## References

[1] UNAIDS (2009). 2009 AIDS Epidemic Update..

[2] World Health Organisation (2008). World Health Statistics 2008..

[3] World Bank (2008). World Development Report..

[4] Ministry of Health Vietnam (2009). Vietnam HIV/AIDS Estimates and Projections 2007-2012..

[5] Ministry of Health Vietnam (2005). HIV/AIDS Estimates and Projections: 2005-2010..

[6] UNAIDS (2008). Report on the Global AIDS Epidemic, 2008.

[7] Nguyen TA, Fylkesnes K, Bui DT, Nguyen TH, Nguyen TL (2007). Human immunodeficiency virus (HIV) infection patterns and risk behaviours in different population groups and provinces in Viet Nam.. Bull World Health Organ.

[8] Denison JA, O'Reilly KR, Schmid GP, Kennedy CE, Sweat MD (2008). HIV voluntary counseling and testing and behavioral risk reduction in developing countries: a meta-analysis, 1990—2005.. AIDS Behav.

[9] UNAIDS (2001). The impact of voluntary counseling and testing: a global review of the benefits and challenges..

[10] Obermeyer CM, Osborn M (2007). The utilization of testing and counseling for HIV: a review of the social and behavioral evidence.. Am J Public Health.

[11] Socialist Republic of Vietnam (2004). Decision of the Prime Minster approving the National Strategy on HIV/AIDS prevention and control in Viet Nam until 2010 with a vision to 2020..

[12] World Health Organisation (2010). World Health Statistics 2010..

[13] National Institute of Hygiene and Epidemiology (NIHE), Vietnam (2006). Results from the HIV/STI Integrated Biological and Behavioral Surveillance (IBBS) in Vietnam: 2005-2006..

[14] Tam VV, Pharris A, Thorson A, Alfven T, Larsson M (In press). "It's not that I forget, it's just that I don't want other people to know": Barriers to and strategies for adherence to antiretroviral therapy among HIV patients in Northern Vietnam..

[15] Moreno S, Mocroft A, Monforte A (2010). Medical and societal consequences of late presentation.. Antivir Ther.

[16] Lanoy E, Mary-Krause M, Tattevin P, Perbost I, Poizot-Martin I (2007). Frequency, determinants and consequences of delayed access to care for HIV infection in France.. Antivir Ther.

[17] Carrizosa CM, Blumberg EJ, Hovell MF, Martinez-Donate AP, Garcia-Gonzalez G (2010). Determinants and prevalence of late HIV testing in Tijuana, Mexico.. AIDS Patient Care STDS.

[18] Deblonde J, De Koker P, Hamers FF, Fontaine J, Luchters S (2010). Barriers to HIV testing in Europe: a systematic review.. Eur J Public Health.

[19] Girardi E, Sabin CA, Monforte AD (2007). Late diagnosis of HIV infection: epidemiological features, consequences and strategies to encourage earlier testing.. J Acquir Immune Defic Syndr.

[20] Thorson A, Hoa NP, Long NH, Allebeck P, Diwan VK (2004). Do women with tuberculosis have a lower likelihood of getting diagnosed? Prevalence and case detection of sputum smear positive pulmonary TB, a population-based study from Vietnam.. J Clin Epidemiol.

[21] Rutstein S, Johnson K (2004). The DHS Wealth Index..

[22] Hosmer DW, Hjort NL (2002). Goodness-of-fit processes for logistic regression: simulation results.. Statistics in Medicine.

[23] Lemeshow S, Hosmer DW (1982). A review of goodness of fit statistics for use in the development of logistic regression models.. Am J Epidemiol.

[24] General Statistics Office Vietnam, National Institute of Hygiene and Epidemiology Vietnam, ORC Macro (2006). Vietnam Population AIDS Indicator Survey (VPAIS): 2005..

[25] Thailand National Statistical Office (2006). Thailand Multiple Indicator Cluster Survey December 2005- February 2006, Final Report..

[26] Oosterhoff P, Hardon AP, Nguyen TA, Pham NY, Wright P (2008). Dealing with a positive result: routine HIV testing of pregnant women in Vietnam.. AIDS Care.

[27] Nguyen TA, Oosterhoff P, Ngoc YP, Wright P, Hardon A (2008). Barriers to access prevention of mother-to-child transmission for HIV positive women in a well-resourced setting in Vietnam.. AIDS Res Ther.

[28] Hong KT, Nguyen TVA, Ogden J (2004). "Because this is the disease of the century": Understanding HIV and AIDS-related stigma and discrimination in Vietnam..

[29] Dinh T-H, Detels R, Nguyen MA (2005). Factors associated with declining HIV testing and failure to return for results among pregnant women in Vietnam.. AIDS.

[30] Filippi V, Ronsmans C, Campbell OM, Graham WJ, Mills A (2006). Maternal health in poor countries: the broader context and a call for action.. Lancet.

[31] Nguyen AT, Nguyen TTH, Vu TBD, Pham HT, Nguyen TL (2008). Household survey in two provinces in Viet Nam estimates HIV prevalence in an urban and a rural population.. AIDS Res Hum Retroviruses.

[32] Chamratrithiron A, Kittisuksathit S, Podhisidha C, Isarabhakdi P, Sabaiying M (2007). National Sexual Behavior Survey of Thailand, 2006..

[33] Makwiza I, Nyirenda L, Bongololo G, Banda T, Chimzizi R (2009). Who has access to counseling and testing and anti-retroviral therapy in Malawi - an equity analysis.. Int J Equity Health.

[34] Schur CL, Berk ML, Dunbar JR, Shapiro MF, Cohn SE (2002). Where to seek care: an examination of people in rural areas with HIV/AIDS.. J Rural Health.

[35] Monjok E, Smesny A, Mgbere O, Essien EJ (2010). Routine HIV Testing in Health Care Settings: The Deterrent Factors to Maximal Implementation in Sub-Saharan Africa.. J Int Assoc Physicians AIDS Care (Chic Ill).

[36] Castelli F, Pietra V, Diallo I, Schumacher RF, Simpore J (2010). Antiretroviral (ARV) Therapy in Resource Poor Countries: What do we Need in Real Life?. Open AIDS J.

[37] Thorson A (2003). Equity and Equality- case detection of Tuberculosis among women and men in Vietnam..

[38] Helleringer S, Kohler HP, Frimpong JA, Mkandawire J (2009). Increasing uptake of HIV testing and counseling among the poorest in sub-Saharan countries through home-based service provision.. J Acquir Immune Defic Syndr.

[39] Piot P, Greener R, Russell S (2007). Squaring the circle: AIDS, poverty, and human development.. PLoS Med.

[40] Nguyen TA, Oosterhoff P, Pham YN, Hardon A, Wright P (2009). Health workers' views on quality of prevention of mother-to-child transmission and postnatal care for HIV-infected women and their children.. Hum Resour Health.

[41] Kalichman SC, Simbayi LC (2003). HIV testing attitudes, AIDS stigma, and voluntary HIV counselling and testing in a black township in Cape Town, South Africa.. Sex Transm Infect.

[42] Chuc NT, Diwan V (2003). FilaBavi, a demographic surveillance site, an epidemiological field laboratory in Vietnam.. Scand J Public.

